# Missing in action: the genetic mysteries of extremely low HDL cholesterol

**DOI:** 10.3389/fcvm.2025.1553259

**Published:** 2025-05-22

**Authors:** Shoshi Sphitzen, Mordechai Golomb, Mohammad Mowaswes, Refael Bitzur, Smadar Horowitz Cederboim, Ronen R. Leker, Marc Gotkine, Itai Chovers, Daniel Schurr, Eran Leitersdorf, Ronen Durst

**Affiliations:** ^1^ Lipid Clinic and Center for Cardiovascular Precision Medicine, Hadassah Hebrew University Medical Center, Jerusalem, Israel; ^2^ Cardiology Department, Hadassah Hebrew University Medical Center, Jerusalem, Israel; ^3^ The Bert W. Strassburger Lipid Center, Sheba Medical Center, Ramat Gan, Israel; ^4^ Department of Neurology, Hadassah Hebrew University Medical Center, Jerusalem, Israel; ^5^ Ophthalmology Department, Hadassah Hebrew University Medical Center, Jerusalem, Israel

**Keywords:** HDL-C, tangies, LCAT, ABCA1, apoA1

## Abstract

**Introduction:**

High-Density Lipoprotein Cholesterol (HDL-C) plays a pivotal role in cardiovascular health, acting as a key component in lipid transport and atheroprotection. While low HDL-C levels in the general population are often the result of multifactorial causes, extremely low HDL-C levels (<20 mg/dl) are rare and may be attributed to underlying genetic defects. Mutations in genes such as *LCAT*, *APOA1*, and *ABCA1*—although exceedingly rare—have been linked to profound alterations in lipid metabolism, often resulting in significant morbidity and increased cardiovascular risk.

**Methods:**

In this study, we used exome sequencing on patients with very low HDL-C.

**Results:**

We identified three patients with pathogenic mutations associated with genetic low HDL-C syndrome, including *ABCA1* [NM_005502.4(ABCA1):c.4175 + 1G > T, chr:9 91757308° C > A, rs375247413], LCAT [NM_000229.2(LCAT):c.349G > A p.Ala117Thr, rs28940886], and *APOA1* [NM_000039.3(APOA1):c.388A > T, p.Lys130*].

**Discussion:**

Each case presented a unique spectrum of clinical phenotypes, systemic complications, and biochemical abnormalities, illustrating the diverse impact of these genetic mutations. We provide a detailed analysis of the clinical and biochemical profiles of these patients, highlighting key aspects of disease manifestation and progression. This report underscores the importance of recognizing and characterizing rare genetic causes of low HDL-C, which may have profound implications for patient care and risk stratification.

## Introduction

High-Density Lipoprotein (HDL-C) cholesterol plays a vital role in promoting cardiovascular health through its central function in lipoprotein metabolism. Severe HDL-C deficiency (<20 mg/dl) in the absence of secondary causes is extremely rare. Patients with such a marked deficiency are prone to early onset atherosclerotic disease and other systemic complications (1). HDL-C's primary function is to remove excess cholesterol from peripheral tissues through reverse cholesterol transport (RCT), delivering it to the liver, organs with high cholesterol needs, or exchanging it with apoB particles like low-density lipoprotein (LDL-C) for disposal. Cholesterol is transported to the liver and steroidogenic tissues via HDL-C's binding to the scavenger receptor B1 (*SR-B1*) and interacting with ATP-dependent transmembrane transporters, ATP-Binding Cassette Transporter A1 (*ABCA1*) and ATP-binding cassette sub-family G member 1 (*ABCG1*), which are highly expressed in tissues such as macrophages, adipose tissue, and the liver (2). HDL-C's core component, apolipoprotein A1 (apoA1), is synthesized in the liver and intestine, and through lipidation, nascent HDL-Cs mature into α-HDL-C, which undergoes constant remodelling. Genetic mutations in genes such as *apoA1*, *LCAT*, and *ABCA1* can lead to severe HDL-C deficiency (3, 4). Partial HDL-C deficiency, or hypoalphalipoproteinemia, defined as plasma HDL-C levels below the 10th percentile, is a major risk factor for coronary heart disease and stroke.

Located in a tertiary referral center, our lipid clinic provides genetic counseling for lipid disorders for Israel. Of the cases referred to us for genetic evaluation we describe three cases of genetically determined HDL-C deficiency in the Israeli population and discuss the importance of identifying such patients. Our routine laboratory tests are done early morning after an overnight fast using the Roche COBAS^TM^ system. LDL-C values are calculated.

## Case reports

### Case 1: tangier disease in an Ashkenazi Jewish family

Tangier disease is an extremely rare genetic disorder caused by mutations in the *ABCA1* gene. It is characterized by severe plasma deficiency or absence of HDL-C and apolipoprotein A-I (apoA-I), leading to the accumulation of cholesteryl esters in various tissues throughout the body. A classic finding on physical examination is the presence of orange-colored tonsils. The disease results in reduced cholesterol efflux from peripheral cells (5–7).

A 77-year-old woman of Ashkenazi Jewish ancestry with a history of consanguinity was diagnosed with Tangier disease in 1997. She presented with syringomyelia-like syndrome (8) and abnormally low levels of HDL-C, LDL-C, and total cholesterol.

**Figure 1:**
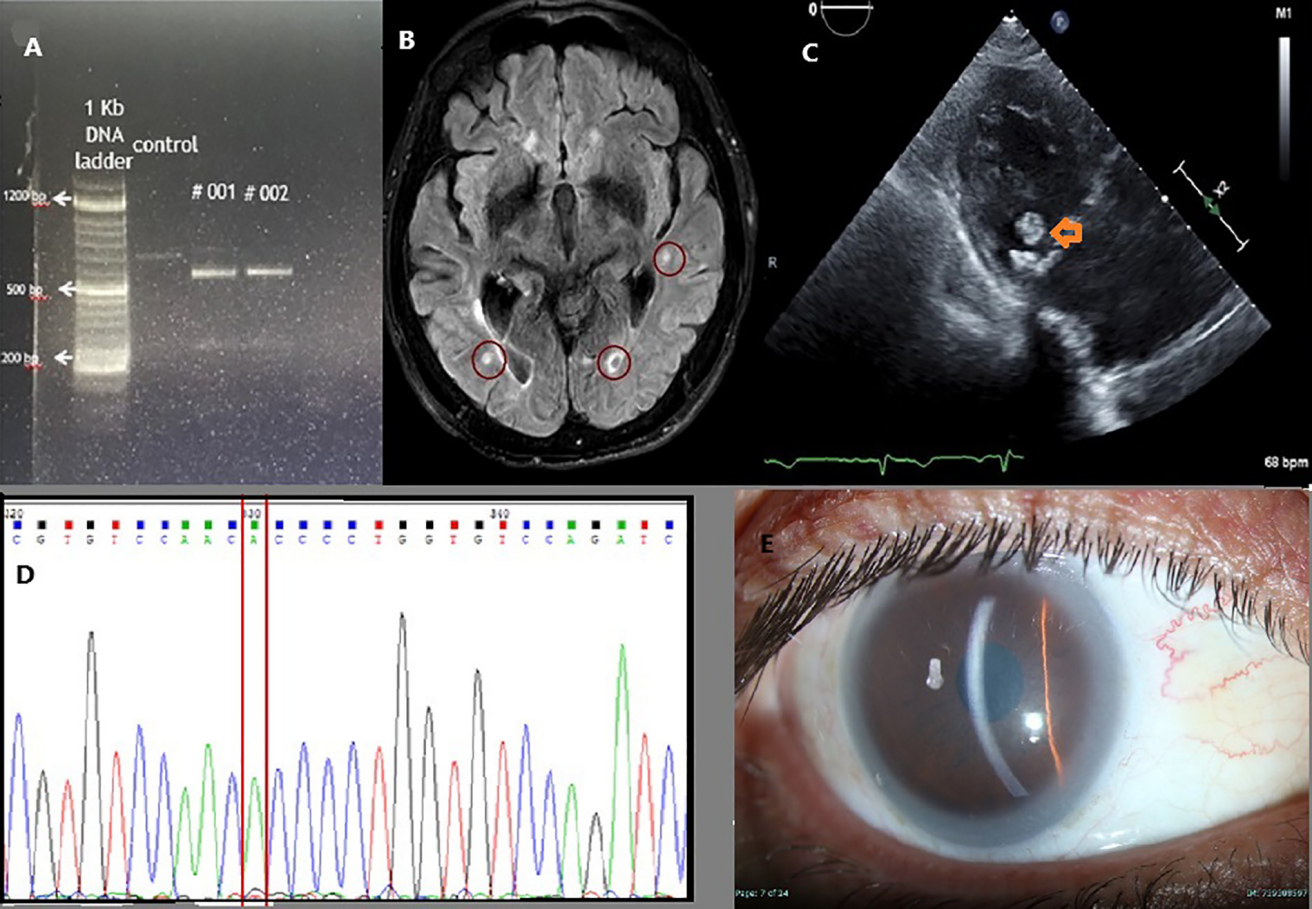
**(A)** Polymerase chain reaction (PCR) products of cDNA extracted from the patient lymphoblasts, spanning the adjacent exons of rs375247413 in the *ABCA1* gene are presented. Lane 1 is control, and the two Tangier Diseased sisters in Lanes #1 & #2. Lower molecular weight fragments in Tangier Disease patients confirm a splice site at the *ABCA1* gene, resulting in shorter fragments due to exon skipping in these. **(B)** MRI FLAIR imaging showing multiple bilateral emboli (red circles). **(C)** Echocardiography with a para-sternal long axis view of the heart. A mobile mass swinging on the posterior leaflet of the mitral valve (orange arrow) suggests a probable cholesterol vegetation. **(D)** Sequencing of the *LCAT* gene demonstrating homozygous splice site error at position 349 substituting G to A. **(E)** Slit lamp picture showing corneal opacities and lipemic arch in the patient with apoA1 deficiency.

Genetic testing through exome sequencing identified a homozygous splice site sequence variation in the *ABCA1* gene [NM_005502.4(ABCA1):c.4175 + 1G > T, chr:9 91757308° C > A, rs375247413, minor allele frequency 0.0000134] Subsequently, a CDNA library was prepared from lymphoblasts of the propositus. PCR flanking the splice variation demonstrated the skipping of the exon downstream of the sequence variation, confirming its pathogenicity (Figure 1A). Her sister was homozygote for the same sequence variation (Figure 1A). Over the past 10 years, the patient's lipid profile has been consistently abnormal (Table 1).

**Table 1:** Cholesterol panel of the three patients.

Plasma apo and lipoprotein	*ABCA1*	*LCAT* mutation	*apoA1* mutation	Normal values range
Triglycerides	217–295	454	97	30–150
Total cholestereol	85–102	88	96	80–200
HDL cholesterol	5–9	10	5	>40
LDL cholesterol	20–47	62	71	
Apolipoprotein A1		65		100–200
Apolipoprotein B		56		40–125
Apolipoprotein C2		7		3–7
Apolipoprotein C2		17.9		9–19

Notably, during her clinical follow-up, a dense, mobile mass measuring 11 × 9 mm was identified on the mitral valve, suggestive of cholesterol accumulation (Figure 1C). This finding correlated with recurrent strokes, emphasizing the cardiovascular implications of Tangier disease. Her sibling had a milder form of the disease. Although she experienced peripheral neuropathy, it was not severe enough to require wheelchair assistance. She had a cerebrovascular accident (CVA), but it was not debilitating. Worth mentioning that she underwent a tonsillectomy during childhood. Interestingly, during a colonoscopy, her mucosa appeared orange, resembling the description of orange tonsils seen in Tangier disease.

The risk of cerebrovascular disease in Tangier's patients was described previously (Figure 1B) (9). Of note, her sister had a CVA episode later on during follow up. A prior report of early onset valve disease as well as our report may suggest valve involvement with Tangier's disease (10).

### Case 2: lecithin-cholesterol acyltransferase (*LCAT*) deficiency

*LCAT* deficiency, also known as Fish Eye Disease (FED), is a rare recessive genetic disorder with only a few cases reported worldwide (11–14). The condition arises due to mutations in the *LCAT* gene, resulting in a lack of lecithin-cholesterol acyltransferase, an enzyme critical for HDL-C formation (11). Clinical manifestations include corneal opacifications (“boiled fish eye”) and dyslipidemia characterized by low HDL-C levels.

A 64-year-old male with a known history of dyslipidemia presented with new-onset visual impairment. Clinical examination revealed corneal opacifications, which raised the suspicion of FED. Genetic testing identified a missense variation [NM_000229.2(LCAT):c.349G > A, p.Ala117Thr, rs28940886, minor allele frequency 0.0000057] (Figure 1D) in the LCAT gene. This change, causing alanine to threonine amino acid substitution in exon 3, was previously documented as pathogenic (15) due to its association with reduced enzyme activity. A detailed lipid profile of this patient is presented in Table 1.

### Case 3: apoA1 deficiency

apoA1 deficiency is a rare disorder of lipoprotein metabolism characterized by the complete absence of apolipoprotein A1, leading to extremely low levels of plasma HDL-C cholesterol. This condition can manifest with significant clinical features, including corneal opacities, xanthomas, and premature coronary heart disease (CHD).

We report a case involving a 30-year-old male proband of Muslim ancestry, who is the only child of first-cousin parents, though he has healthy half-siblings from both sides. He has three healthy daughters aged 17 to 2. The patient experienced a myocardial infarction at the age of 33, which prompted further investigation revealing corneal lipid deposits compatible with systemic hyperlipidemia (as shown in Figure 1E) and undetectable plasma HDL-C cholesterol levels. Genetic analysis confirmed that he was homozygous for the novel *apoA1* [NM_000039.3(APOA1):c.388A > T, p.Lys130*, novel mutation] single nucleotide change, resulting in the premature termination of apolipoprotein A1 translation on exon 4. This change is novel, not reported before. It is likely pathogenic because of the early termination codon. Over a 10-year follow-up period, he underwent five coronary revascularization procedures, underscoring the severe cardiovascular consequences associated with apoA1 deficiency and highlighting the urgent need for awareness and management strategies for affected individuals.

## Discussion

HDL-C cholesterol facilitates reverse cholesterol transport from peripheral tissues to the liver, playing a crucial role in cardiovascular health. Genetic factors significantly influence HDL-C cholesterol levels, affecting the body's ability to maintain this essential biological function. Cholesterol ester transfer protein (*CETP*), *LCAT*, and *ABCA1* are critical genes involved in regulating HDL-C levels (2). Several genetic mutations have been identified that contribute to low HDL-C cholesterol levels, each associated with distinct clinical phenotypes and consequences. Alves et al. (16) presented cases of rare dyslipidemias and in cases with reduced HDL-C levels, they defined mutation in either the *ABCA1*, *apoA1*, and *LCAT* genes.

Similarly, we present three cases of genetically determined HDL-C deficiency associated with pathogenic DNA sequence variation in the *ABCA1*, *LCAT*, and *apoA1* genes. The diverse mutations, along with their varying vascular and extravascular phenotypes, highlight the importance of modern genetic testing in determining the underlying genetic cause. Early-onset atherogenic disease was a common feature across all three cases, while the extravascular manifestations differed depending on the specific mutated gene. Although the phenotype may provide clues to the genetic cause of the disease, genotyping is crucial in most cases -especially those with predominantly vascular phenotypes -for accurately identifying the cause of low HDL-C and predicting potential future organ system involvement. In Tangier disease, clinical manifestations may include a highly atypical neuropathy, mimicking syringomyelia or leprosy (8), and orange discoloration of lymphatic tissues (6). Notably, we describe the first novel splice site error in the *ABCA1* gene within an Ashkenazi Jewish population, where a tendency toward consanguinity suggests that other undiagnosed cases of Tangier disease may exist (17). Additionally, the presence of cholesterol deposits on the mitral valve and their association with stroke further delineate the systemic consequences of cholesterol dysregulation. The cases of LCAT and apoA1 deficiencies demonstrate the diverse phenotypic spectrum of HDL-C-related metabolic disorders, highlighting how early genetic screening can guide clinical management and follow-up strategies. A significant manifestation in these patients is corneal opacification, which serves as a critical diagnostic feature (13). The *CETP* gene encodes a protein that transfers cholesteryl esters and triglycerides between lipoproteins. LCAT is responsible for the esterification of free cholesterol on HDL-C particles, a key step in HDL-C maturation. *ABCA1* mediates the efflux of cholesterol and phospholipids to apolipoprotein AI, forming nascent HDL-C particles. The role of *ABCA1* actually extends beyond lipid efflux, including plasma membrane remodeling and apoAI binding (18, 19).

Clinical management of individuals with genetically low HDL-C cholesterol levels requires a nuanced approach. Lifestyle modifications remain fundamental but have negligible effects on HDL-C levels. Similarly, reducing traditional risk factors, such as lowering LDL cholesterol and physical activity, has a very limited impact on HDL-C metabolism. Unfortunately, pharmacological interventions designed to raise HDL-C levels have yielded negative results in large clinical trials with CETP inhibitors and niacin (20, 21). Trials with HDL-C mimetics in patients with genetically determined very low HDL-C have also failed to show clinical benefit, despite significant increases in cholesterol efflux among treated patients (22). Thus, the current state of the art is early diagnosis of HDL-C deficiency syndrome patients and targeting modifiable risk factors. Recent advances in genetic research focus on gene therapy and CRISPR technologies. For example, researchers have used AAV vector to deliver the human ApoA-I gene in mice subjected to cardiac pressure overload. Several beneficial cardiovascular outcomes were demonstrated including reduced septal wall thickness, improved myocardial capillary density, and reduced interstitial cardiac fibrosis (23). Such technologies may, in the future, strengthen our ability to treat genetic HDL-C deficiency; however, these disorders currently do not have targeted treatment.

## Summary

This report shares our experience in a tertiary referral centre in identifying and treating genetically determined very low HDL-C syndrome. We described three patients with mutations in *CETP*, *LCAT*, and ABCA1, demonstrating the clinical presentation and risk for cardiovascular disease. There remains an unmet need for effective treatments for this condition, as targeted therapies have thus far failed to show clinical benefit.

## Data Availability

The datasets presented in this study can be found in online repositories. The names of the repository/repositories and accession number(s) can be found below: https://www.ncbi.nlm.nih.gov/snp/, rs375247413.
